# Baricitinib Liposomes as a New Approach for the Treatment of Sjögren’s Syndrome

**DOI:** 10.3390/pharmaceutics14091895

**Published:** 2022-09-07

**Authors:** Núria Garrós, Mireia Mallandrich, Negar Beirampour, Roya Mohammadi, Òscar Domènech, Maria José Rodríguez-Lagunas, Beatriz Clares, Helena Colom

**Affiliations:** 1 Department of Pharmacy, Pharmaceutical Technology and Physical Chemistry, Faculty of Pharmacy and Food Sciences, University of Barcelona, 08028 Barcelona, Spain; 2 Institute of Nanoscience and nanotechnology, University of Barcelona, 645 Diagonal Avenue, 08028 Barcelona, Spain; 3 Department of Physiology, Faculty of Pharmacy and Food Sciences, University of Barcelona, Av. Joan XXIII, 08028 Barcelona, Spain; 4 Department of Pharmacy and Pharmaceutical Technology, Faculty of Pharmacy, University of Granada, 18071 Granada, Spain

**Keywords:** Sjögren’s syndrome, liposomes, baricitinib, JAK inhibitor, ocular delivery, transcorneal permeation, transscleral permeation, ocular tolerance

## Abstract

Sjögren’s syndrome is a chronic systemic autoimmune disease affecting from 0.2 to 3% of the general population. The current treatment for Sjögren’s syndrome is aimed at controlling symptoms such as dry eyes and xerostomia. Systemic therapy with glucocorticoids or immunosuppressants is also used. Baricitinib is an immunosuppressant drug, specifically a Janus kinases 1 and 2 selective inhibitor. We propose ocular liposomal formulations loaded with baricitinib for the management of Sjögren’s syndrome. The novelty of the work relies on the fact that, for the first time, baricitinib is intended to be used for topical delivery. Two liposomal formulations were prepared with different lipids: (i) L-α-phosphatidylcholine (Lα-PC) and (ii) a combination of lipids 1-palmitoyl-2-oleoyl-phosphatidylethanolamine: s1-Palmitoyl-2-oleoyl-sn-glycerol-3-phosphoglycerol (3:1, mol/mol) (POPE:POPG), and they were physicochemically characterized. The in vitro drug release and the ex vivo permeation through corneal and scleral tissues were also assessed. Finally, the tolerance of the formulations on the ocular tissues was evaluated by the HET-CAM technique, as well as through the histological analysis of the cornea and sclera and the cornea transparency. Both liposomes resulted in small, spherical shapes, with suitable physicochemical properties for the ocular administration. Lα-PC led to higher flux, permeation, and retention in the sclera, whereas POPE:POPG led to higher flux and permeation in the cornea. The formulations showed no irritant effects on the chorioallantoic membrane. Additionally, the liposomes did not affect the cornea transparency when they were applied, and the histological analysis did not reveal any structural alteration.

## 1. Introduction

Immunologic diseases are caused by an imbalance between the immune system function to protect the body from bacteria and viruses and tissue damage because of the immune response [1]. Some kinds of autoimmune diseases are immunologic diseases, which are the result of identifying the patient’s own organs, tissues, and cells as foreign and activating an immune response against them. Individually, these diseases are rare, but as a group, they are the most common diseases in industrialized countries [2], and they affect between 5 and 10% of the European and North American population [3].

Sjögren’s syndrome (SS) is a chronic systemic autoimmune disease that can be suffered as a unique disease (primary SS) or can be a consequence of another autoimmune disease (secondary SS) [4]. Primary SS is a highly prevalent, chronic, autoimmune exocrinopathy today, affecting from 0.2 to 3% of the general population. It is caused by the loss of central tolerance, which generates epithelitis and acinar atrophy due to predominantly inflammatory cell types which infiltrate into the exocrine glands and certain extraglandular tissues [5]. It is characterized by keratoconjunctivitis sicca (dry eyes) and xerostomia (dry mouth) [4,6].

The European Study Group on Classification Criteria developed and validated a criteria classification for primary SS between 1989 and 1996. It was made observing 180 people, of which 76 were affected by primary SS and 104 were not. It is organized in a classification tree performance and has a sensitivity of 96.1% and a specificity of 94.2% [7].

Different anti-inflammatory and immunomodulatory drugs are used in the treatment of ocular inflammatory and immunological diseases. Two examples are diclofenac, used as an anti-inflammatory agent, and cyclosporine, used an as anti-inflammatory and immunomodulatory drug [8]. Baricitinib is an immunosuppressant drug that acts inhibiting selectively Janus kinases 1 and 2, among others, and reduces disease signs and symptoms by decreasing inflammation, cellular activation, and proliferation of key immune cells. Baricitinib has already been used for the treatment of atopic dermatitis through oral administration with good outcomes: oral baricitinib improved signs and resolved symptoms better than topic cyclosporin [9]. Another advantage is that baricitinib has anti-inflammatory properties due to its therapeutic path. Now, baricitinib is orally administrated for the treatment of moderate-to-severe atopic dermatitis and rheumatoid arthritis, and it is also being studied for its oral administration for systemic erythematosusmatous, psoriasis, and primary SS [10,11]. The pilot study of baricitinib oral administration conducted in China using active SS patients seemed to show efficacy and safety [12]. The actual treatment for primary SS is divided into topical treatment for the mouth and eyes to control the symptoms and avoid complications, and systemic treatment for parotid enlargement and extraglandular signs. Topical treatments are preventive: fluor is used to avoid periodontal diseases and chlorhexidine for electrostimulation; and for the eyes, artificial tears are used. Anti-inflammatory drugs and local ciclosporin, pilocarpine, or cevimeline secretagogue are used in both cases to stimulate saliva or lacrimal flow. Systemic treatments consist of glucocorticoids and different immunosuppressants [13].

**Figure 1:**
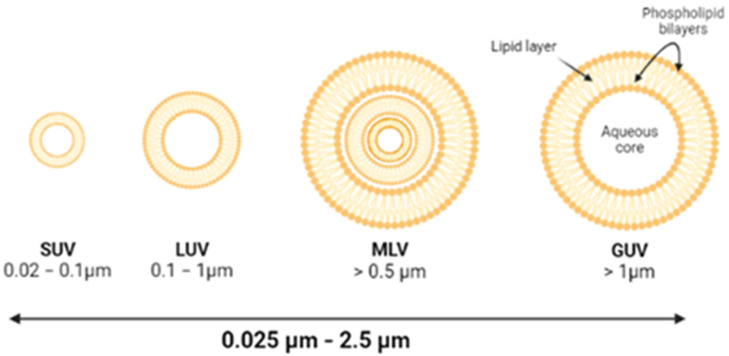
Classification of the liposomes based on their size and number of lipid layers. SUV, small unilamellar vesicle; LUV, large unilamellar vesicle; MLV, multilamellar liposome vesicle; GUV, giant unilamellar vesicle.

Liposomes are spherically shaped nano-sized to micro-sized vesicles composed of biodegradable natural or synthetic phospholipids. They are formed spontaneously in an aqueous medium so that inside the vesicles different agents can be encapsulated; the hydrophobic agents between the lipids and the hydrophilic ones in the aqueous core [14]. Liposomes can have very different properties because of their composition, surface charge, size, phospholipid bilayer membrane, and method of preparation [15,16,17]. One way for liposomes to be classified is by their size and number of bilayers: multilamellar liposome vesicles (MLV) and unilamellar vesicles (UV). The latter group has three more stages, as shown in Figure 1.

The liposomes’ composition is biocompatible, biodegradable, and non-toxic, and the flexibility of their formulation allows for different sizes of liposomes that make it possible to use them as eye drops [8]. These characteristics also help at the time of preparing an ophthalmic formulation, during which, some points need to be studied to avoid compatibility problems: the pH has to be in a range close to the physiologic pH, the osmolarity has to be isotonic with the tears and to prevent infections, and sterility is also necessary [18]. The high capacity of entrapment relies on its ability to encapsulate a wide range of drugs, and having this advantage overcomes the common problem of drugs that are water-insoluble or poorly soluble in this kind of formulation [19]. As for liposome size and charge for eye application, it has been shown that the interaction of liposomes with cornea follow this order: MLV^+^ > SUV^+^ > MLV^−^ > SUV^−^ > MLV [8].

To our knowledge, no studies involving baricitinib administered by the ophthalmic route have been conducted. Stevenson and co-workers determined the efficacy of another Janus kinase inhibitor; 0.003% tofacitinib was applied ocularly in mice which had induced corneal thermocautery, resulting in a decrease in the interleukins [20]. In another study, Hofauer and colleagues investigated the efficacy of liposomal agents for the symptoms of xerostomia, keratoconjunctivitis sicca, and rhinitis sicca in a clinical trial involving patients with Sjögren’s syndrome. The authors concluded that liposomes were an effective local approach since they significantly reduced the symptoms of xerostomia, keratoconjunctivitis sicca, and rhinitis sicca after 2 months of treatment [21]. Taking into account the satisfactory results obtained by Stevenson et al. with tofacitinib and those obtained by Hofauer with liposomal agents, we aimed to formulate liposomes loading baricitinib for ophthalmic administration as an alternative or co-adjuvant treatment for Sjögren’s syndrome [9]. Therefore, our intention with these formulations is to simplify the eye topical treatment to one step. We characterized two liposomes with two different lipids and we assessed the baricitinib release from the formulations as well as the capacity of baricitinib to penetrate the corneal and scleral tissues through ex vivo permeation tests. We also investigated the tolerability of the developed formulations on the eye by alternative in vitro methods, such as the HET-CAM technique and the evaluation of the cornea transparency. Finally, we conducted histological studies on the tissues after the permeation test.

## 2. Materials and Methods

### 2.1. Materials

Baricitinib and an Ammonium salt formate were bought at Sigma-Aldrich (Madrid, Spain). Gattefossé (Barcelona, Spain) supplied Transcutol^®^ P [Diethylene glycol monoethyl ether]. Acetonitrile was purchased at Fisher Chemical (Loughborough, UK). Lipids L-α-phosphatidylcholine (Lα-PC) and 1-palmitoyl-2-oleoyl-phosphatidylethanolamine (POPE) were bought at BOC Sciences (London, UK) and 1-Palmitoyl-2-oleoyl-sn-glycerol-3-phosphoglycerol (POPG) was obtained at Sigma-Aldrich (Madrid, Spain).

### 2.2. Biological Materials

Cornea and sclera were obtained from residual individuals of female pigs (cross Landrace x Large White, 25–30 kg), previously used in surgical university practices and according to the Ethics Committee of Animals Experimentation at the University of Barcelona. The eyes were immediately enucleated after the animals were sacrificed, and corneas and scleral tissues were excised in situ and transported to the laboratory immersed in artificial aqueous humor solution to be debrided and plain-prepared for the permeation experiments.

### 2.3. Methods

#### 2.3.1. Preparation of the Liposomes

A total of 500 mg of baricitinib was placed in a round-bottom flask and 10 mM chloroform-methanol (2:1, *v*/*v*) lipid solutions -Lα-PC or POPE:POPG (3:1, mol/mol) were added to the flask to obtain the required lipid molar concentration for each composition [22]. To ensure the baricitinib was fully dissolved, the solution was sonicated for 10–15 s. The round-bottom flask was then mounted on a rotary evaporator and the solvent was evaporated protected from the light. Dry lipids with baricitinib were left under a high vacuum in a desiccator protected from light overnight. Thin films were rehydrated with 10 mM TRIS·HCl, 150 mM NaCl pH 7.40 [23,24] supplemented with a 5% (*v*/*v*) of Transcutol^®^ P. Large multilamellar vesicles were obtained after 5 cycles of vigorous vortexing of the solution over and below the transition temperature of the lipid mixture. To homogenize the liposome size, the liposome solution was placed in an ultrasound bath with 100% sonication amplitude (the controlling temperature did not exceed the 37 °C) for 15 min. Finally, non-encapsulated baricitinib was eliminated by filtering the liposomal solution through a Sephadex^®^ G50 column mounted in a 5 mL syringe and centrifuged at 1000× *g* rpm in a Rotanta 460R centrifuge (Andreas Hettich GmbH & Co. KG, Tuttlingen, Germany).

#### 2.3.2. Liposomes Physicochemical Characterization

The physicochemical characterization included the measurement of pH, the vesicle size and polydispersity index, the zeta potential, the osmolality, and the efficiency of encapsulation.

pH was measured at room temperature with pH-metre micro pH 2001 (Crison Instruments SA, Alella, Spain) by triplicate.

Liposome size, polydispersity index (PDI), and zeta potential (ZP) were measured with a Zetasizer Nano S (Malvern Instruments, Malvern, UK); all measurements were made in triplicate and showed satisfactory deviation values [25]. The surface electrical properties of the liposomes were measured after suitable dilution (0.1% *w*/*v*) by electrophoresis measures using a Zetasizer 2000 (Malvern Instruments Ltd., UK). Furthermore, the influences of pH and ionic strength were also investigated. For this task, dilute liposomal dispersions were prepared at different pHs (3–8) and ZP determinations were done after they had been in contact for 24 h under mechanical stirring (50 rpm) and at 25.0 ± 0.5 °C. Before carrying out the measurement, the pH was checked and readjusted. Similarly, the ZP values were also recorded for liposomes formulated at pH 6 with different electrolytes. Hence, the effect of the particular electrolyte (NaCl, CaCl_2_, and AlCl_3_) and concentrations ranging from, 2 × 10^−1^, 10^−1^, 10^−2^, 10^−3^, 10^−4^, and 10^−5^ M, were assayed. All measurements were also performed on blank liposomes and loaded liposomes nine times.

Osmolality was measured using an Advanced 3320 Micro-Osmometer (Advanced Instruments, LLC, Norwood, MA, USA) [26].

The efficiency of encapsulation (*EE*) was measured by breaking the liposomes with 80% of Transcutol^®^ P and a 10% of 10% Triton and quantifying the amount of baricitinib by HPLC. The amount of baricitinib was compared to the initial amount (Equation (1)) [26].

(1) $$EE\%=\frac{Q_{f}}{Q_{o}}\times 100,$$

where, *EE%* is the efficiency of encapsulation, *Q_f_* is the amount of baricitinib in mg retained inside liposomes, and *Q_o_* is the amount of baricitinib in mg used initially to elaborate liposomes.

#### 2.3.3. Morphological Study of the Liposomes

The morphological study was carried out by TEM with a JEM-1010 microscope (JEOL Ltd., Tokyo, Japan). One drop of each liposome was put on copper grids covered with a layer of Formvar^®^. The sample was in contact with the grid for 1 min. Next, one drop of 2% uranyl acetate solution was placed on the grid, and subsequently, a drop of methylcellulose was placed on the grid for 10 min; the excess methylcellulose was wiped with filter paper, tapping the filter diagonally. Finally, the grid was allowed to dry before image analysis [27].

#### 2.3.4. In Vitro Drug Release Study

In vitro release studies were carried out using Franz-type diffusion cells [28,29] with a diffusion area of 0.64 cm^2^ and a receptor chamber of 4.9 mL. We used a dialysis membrane with a molecular cut-off weight of 14,000 Da (Sigma-Aldrich, Madrid, Spain). The membrane was hydrated for 24 h in methanol:water (1:1) and rinsed before being mounted in the Franz diffusion cell (Crown Glass Company, Inc., Jersey City, NJ, USA).

Transcutol^®^ P was the receptor medium which provided the sink conditions throughout the study. Aliquots of 500 μL of two different liposomes were added to the donor compartment. A volume of 200 μL was taken and replaced with Transcutol^®^ P at established times over 31 h. The experiment conditions are set out in Table 1. The samples obtained were analyzed by a validated HPLC-fluorescence method. The concentrations of baricitinib in the liposomes were determined as described in the efficiency encapsulation section. The data were fitted to different kinetic models and the best fit was selected based on the determination coefficient r^2^ [30].

#### 2.3.5. Ex Vivo Permeation Study

Ex vivo corneal and scleral permeation was conducted with Franz diffusion cells. The tissues were fixed between the donor and the receptor compartments [28,29]; the area exposed to permeations was 0.64 cm^2^. We applied 500 μL of liposome, either Lα-PC or POPE:POPG, in the donor compartment, with five replicates for each tissue. The receptor compartment was Transcutol^®^ P kept at 37 °C for the scleral tissue and at 32 °C for the cornea, and stirred continuously. A volume of 200 μL was withdrawn from the receptor compartment at fixed times and replaced by an equivalent volume of Transcutol^®^ P. Experimental conditions for the ex vivo permeation test are shown in Table 2. The samples were quantified by HPLC with a fluorescence detector.

**Figure 2:**
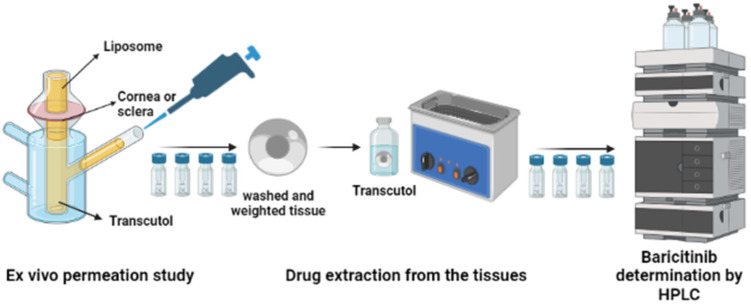
Ex vivo permeation studies diagram.

Once the permeation study was complete, all tissues were removed from the diffusion cells and rinsed with distilled water to eliminate the liposomes remaining on the tissue surface. To extract baricitinib retained in the tissues [25], the permeation area was cut out, weighed, and immersed in 1 mL of Transcutol^®^ P and sonicated for 10 min using an ultrasonic water bath. The supernatant was filtered and quantified by HPLC. Figure 2 depicts the procedure for drug extraction from the corneal and scleral tissues. The amount retained in the tissues (*Qret*) was calculated according to Equation (2), and the results are expressed normalized by the weight of the tissue as well as by the diffusion area (0.64 cm^2^) and multiplied by the recovery of the drug:

(2) $$Qret=\frac{Q_{ext}}{W\times A}\times \frac{100}{R},$$

where, *Q_ext_* is the amount of drug extracted expressed in μg, *W* is the weight of the tissue (g), *A* is the diffusion area (cm^2^), and *R* is the recovery of baricitinib in each tissue [29].

#### 2.3.6. Baricitinib Determination by HPLC

The amount of baricitinib in each sample was quantified by HPLC with a fluorescence detector. The HPLC is composed of a Chromatograph Waters Alliance 2695 and a Fluorescence Jasco FP-1520 detector at an Ex wavelength of 310 nm and an Em wavelength of 390 nm. Table 3 shows the chromatographic conditions for analyzing baricitinib.

#### 2.3.7. In Vitro Tolerance Study

The potential risk of ocular irritation caused by baricitinib liposomes was studied by the HET-CAM test, which measured the ability to induce toxicity on the chorioallantoic membrane (CAM) of a 10-day embryonated hen’s egg (from the G.A.L.L.S.A. farm, Tarragona, Spain). The effects are recorded in seconds during 5 min by the onset of hemorrhage (bleeding), coagulation (blood vessel disintegration), and vessel lysis coagulation (protein denaturation intra- and extra-vascular) [26]. These elements were considered individually and then combined to derive a score (*IS*), which was used to classify the irritancy level of the test substance [31].

(3) $$IS=\frac{301-\sec\;H}{300}\cdot 5+\frac{301-\sec\;L}{300}\cdot 7+\frac{301-\sec\;C}{300}\cdot 9,$$

where, *H* is the hemorrhage, *L* is vessel lysis, *C* is coagulation, and *sec* is the time in seconds when signs started.

We applied 300 μL of liposomes to CAM and we observed the membrane for 5 min to determine the degree of severity of each reaction according to the INVITTOX protocol [32]. We used NaOH 0.1 N as the positive control, and a solution of 0.9% NaCl as the negative control [32].

Additionally, we evaluated changes in the corneal transparency after applying the liposomes to the cornea. The technique consists of exposing the cornea under a defined beam of light and detecting the light transmitted without absorption or scattering [33]. We examined the transmittance from 150 to 760 nm on corneas after these had been immersed in Liposome Lα-PC, Liposome POPE:POPG, PBS pH 7.4 (negative control), and ethanol (positive control) for 10 min [34].

#### 2.3.8. Corneal and Scleral Histological Study

For the histological study of cornea and sclera, samples of both tissues were exposed to the dilution of Liposome Lα-PC, the dilution of Liposome POPE:POPG, or to distilled water (negative control) for 6 h, and then processed for hematoxylin and eosin staining [35]. In a brief summary, corneas and scleral tissues were fixed in 4% buffered paraformaldehyde for 24 h and then, after dehydration, these tissues were embedded in paraffin and cut at 6 µm, stained, and mounted on DPX (Sigma Aldrich). Samples were observed under the microscope (Olympus BX41 and camera Olympus XC50) on a blind coded sample.

## 3. Results

### 3.1. Liposomes Physicochemical Characterization

The pH, osmolality, and encapsulation efficiency of the liposomes were measured and the results are presented in Table 4. Both liposomes showed physiological pH, a suitable osmolality value for the ophthalmic application, and encapsulation efficiency below 20%.

Both liposomes have similar sizes and are negatively charged. Specifically, POPE:POPG exhibits a higher ZP. Values are shown in Table 5.

**Figure 3:**
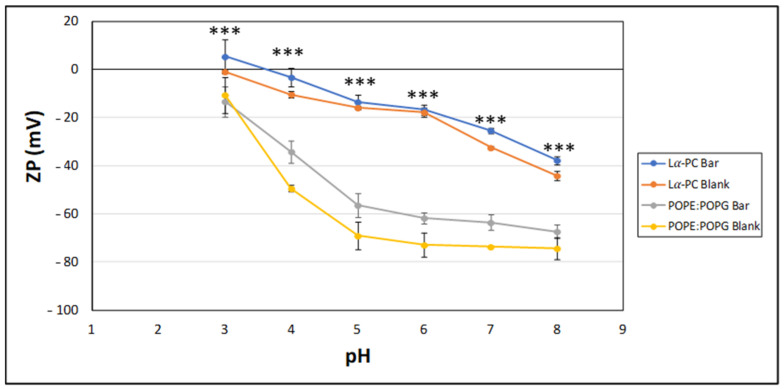
ZP of liposomes as a function of pH. Each point represents the Mean ± SD (n = 9). *** Statistical differences between Lα-PC liposomes and POPE:POPG liposomes (*p* < 0.0001) on a *t*-Test analysis comparing Lα-PC vs. POPE:POPG loaded with baricitinib. Significance level set at *p* < 0.05. Lα-PC = L-α-phosphatidylcholine; POPE:POPG = 1-palmitoyl-2-oleoyl-phosphatidylethanolamine: 1-Palmitoyl-2-oleoyl-sn-glycerol-3-phosphoglycerol (3:1, mol/mol).

The surface electrical study revealed that liposomes showed dependence on the pH. The ZP values revealed the negative surface charge of the liposomes in the entire pH range for all formulations, and no differences between blank liposomes and loaded liposomes were observed. Figure 3 shows the zeta potential of the liposomes as a function of pH.

**Figure 4:**
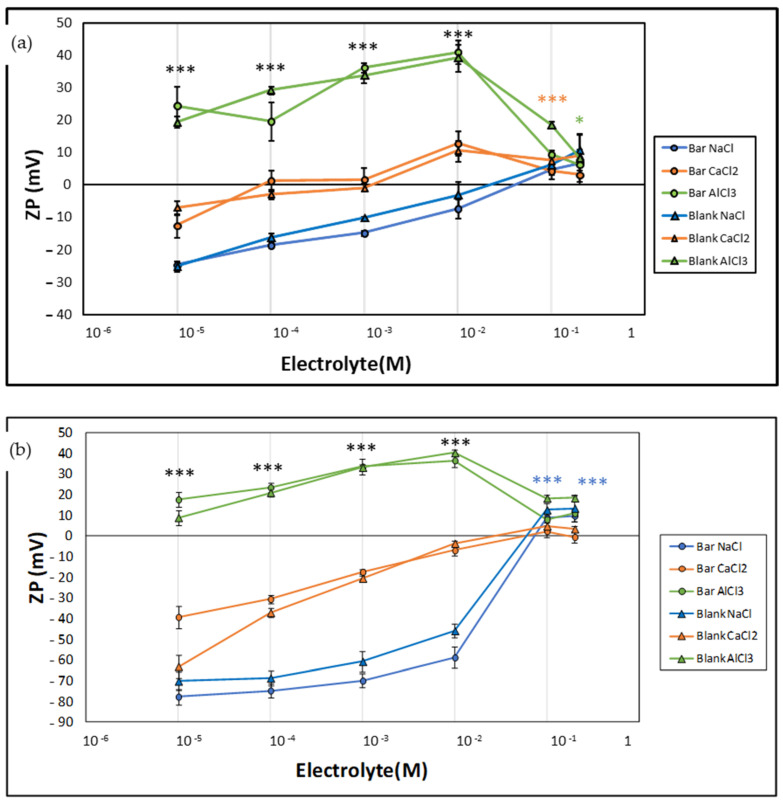
ZP of liposomes as a function of the concentration of NaCl, CaCl_2_, and AlCl_3_ at pH 7.4. (**a**) Lα-PC liposomes and (**b**) POPE:POPG liposomes. Each point represents the mean ± SD (n = 9). An ANOVA was conducted and then followed by Tukey analysis, considering three groups of electrolyte types at each concentration for both liposomes Lα-PC and POPE:POPG loading baricitinib. *** Statistical differences *p* < 0.0001 between all the electrolytes; *** Statistical differences *p* < 0.0001 between NaCl vs. AlCl_3_ for Lα-PC; * Statistical differences *p* < 0.05 between NaCl and CaCl_2_ vs. AlCl_3_ for Lα-PC; *** Statistical differences *p* < 0.0001 between NaCl and AlCl_3_ vs. CaCl_2_. Lα-PC = L-α-phosphatidylcholine; POPE:POPG = 1-palmitoyl-2-oleoyl-phosphatidylethanolamine: 1-Palmitoyl-2-oleoyl-sn-glycerol-3-phosphoglycerol (3:1, mol/mol).

The results of ZP as a function of ionic strength are shown in Figure 4. In general, it is observed that the absolute value tended to decrease as the concentration of either electrolyte increased.

Regarding the pH study, no differences between blank liposomes and loaded liposomes were recorded. Specifically, for monovalent and divalent cations, the ZP at low electrolyte concentrations showed negative values and decreasing absolute values as the electrolyte concentration increased. In the case of NaCl, this negativity was maintained throughout the range in the vicinity of 0, and was slightly positive when the salt reached a concentration of 10^−2^ M, 10^−1^ M, and 2 × 10^−1^ M, respectively. In the presence of Ca^2+^ ions, ZP values became positive or close to 0 from 10^−4^ M or 10^−2^ M, depending on the type of liposome. Finally, ZP values were positive throughout the range of concentrations tested for Al^3+^.

### 3.2. Morphological Study of the Liposomes

**Figure 5:**
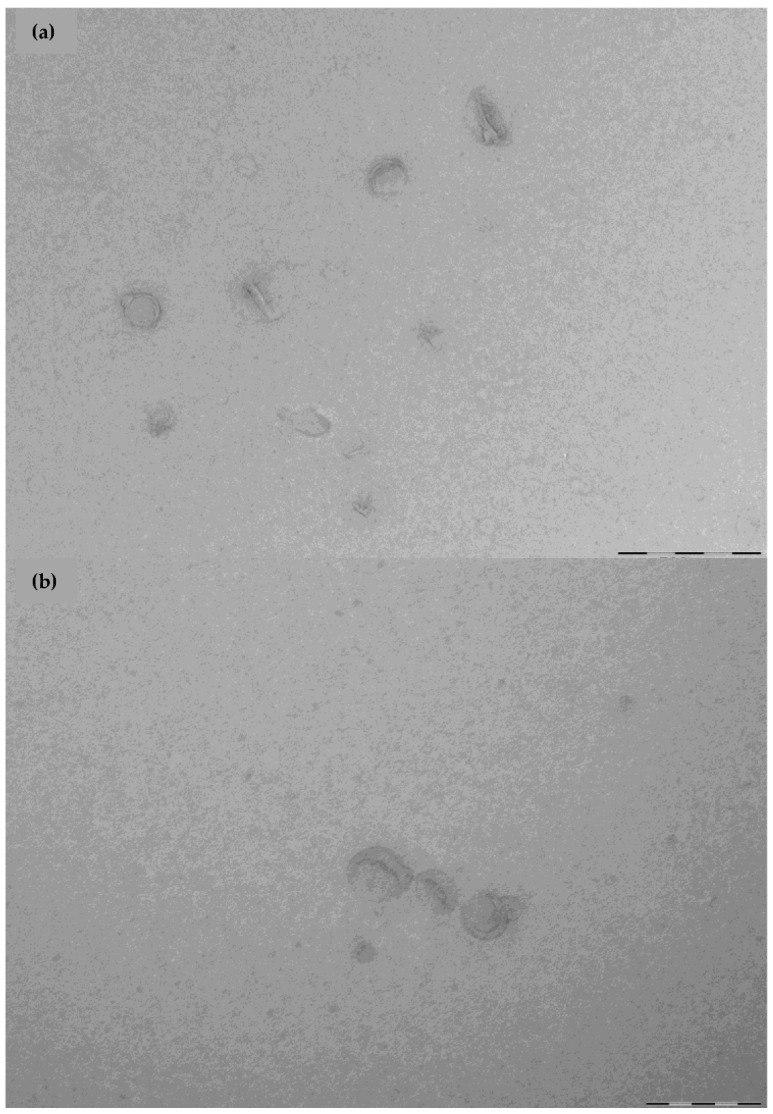
TEM images of the liposomes. (**a**) Liposome Lα-PC (L-α-phosphatidylcholine), and (**b**) POPE:POPG (1-palmitoyl-2-oleoyl-phosphatidylethanolamine: 1-Palmitoyl-2-oleoyl-sn-glycerol-3-phosphoglycerol (3:1, mol/mol). The scale bar stands for 200 nm.

TEM images of both liposomes are shown in Figure 5. The liposomes obtained resulted in a spherical shape and no aggregates were observed.

### 3.3. In Vitro Drug Release Study

**Figure 6:**
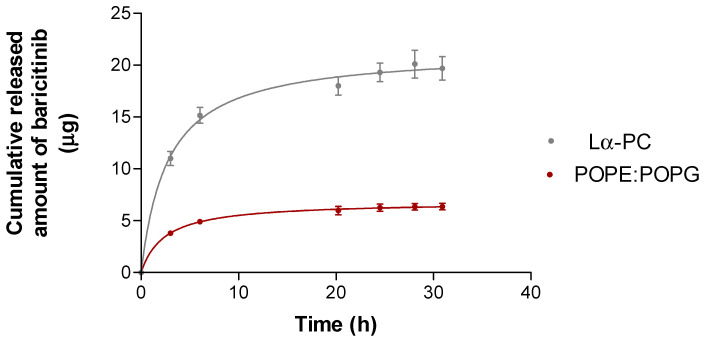
Release profiles of baricitinib from the liposomes POPE:POPG (1-palmitoyl-2-oleoyl-phosphatidylethanolamine: 1-Palmitoyl-2-oleoyl-sn-glycerol-3-phosphoglycerol (3:1, mol/mol), and Lα-PC (L-α-phosphatidylcholine): baricitinib cumulative released (μg) vs. time (h). Results are expressed by mean ± SD (n = 5).

The release profile of both liposomes fits a nonlinear regression. They follow a hyperbola system where the liposome Lα-PC can release the drug faster than liposome POPE:POPG, and in addition, the liposome Lα-PC can release all of the drug, whereas liposome POPE:POPG can release just 64%. The rest remains trapped. Figure 6 shows the release profiles of baricitinib. Both liposomes’ data are fitted to the one-site binding model (Y = Bmax∗X/KD + X). Table 6 shows the fitted values for the model’s parameters Bmax and KD. The statistical analysis by a *t*-Test comparing Lα-PC and POPE:POPG showed significant differences for Bmax.

### 3.4. Ex Vivo Permeation Study

**Figure 7:**
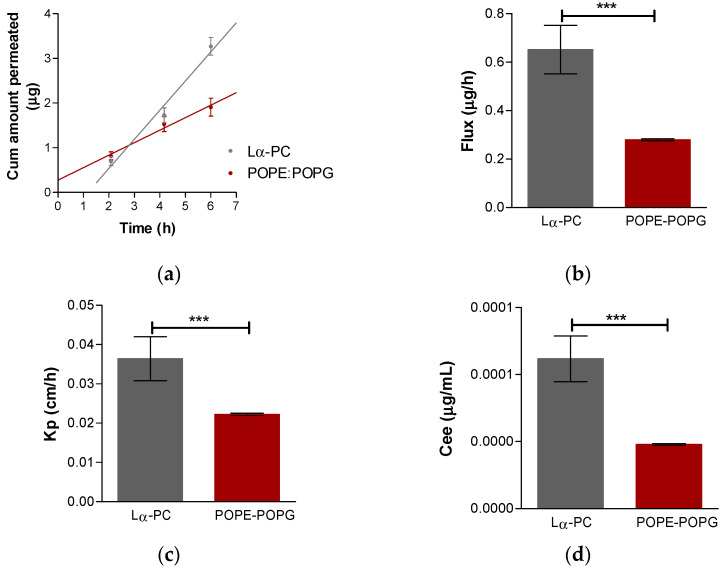
Ex vivo transscleral permeation. (**a**) Baricitinib permeation profile: baricitinib cumulative amount permeated (μg) vs. time (h); (**b**) baricitinib flux for each liposome; (**c**) baricitinib amount retained in the scleral tissue; (**d**) theoretical plasma concentration at the steady-state in humans. Results are expressed by mean ± SD (n = 5). Lα-PC = L-α-phosphatidylcholine; POPE:POPG = 1-palmitoyl-2-oleoyl-phosphatidylethanolamine: 1-Palmitoyl-2-oleoyl-sn-glycerol-3-phosphoglycerol (3:1, mol/mol). *t*-Test analysis with statistically significant difference: *** = *p* < 0.0001.

**Figure 8:**
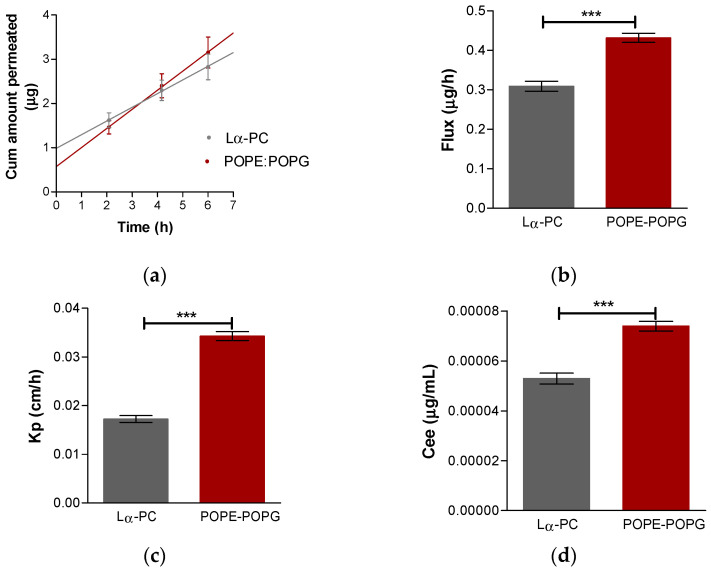
Ex vivo transcorneal permeation. (**a**) Baricitinib permeation profile: baricitinib cumulative amount permeated (μg) vs. time (h); (**b**) baricitinib flux for each liposome; (**c**) baricitinib retained amount in the cornea; (**d**) theoretical plasma concentration at the steady-state in humans. Results are expressed by mean ± SD (n = 5). Lα-PC = L-α-phosphatidylcholine; POPE:POPG = 1-palmitoyl-2-oleoyl-phosphatidylethanolamine: 1-Palmitoyl-2-oleoyl-sn-glycerol-3-phosphoglycerol (3:1, mol/mol). *t*-Test analysis, statistically significant difference: *** = *p* < 0.0001.

Both liposomes on the cornea have r^2^ values of 0.99, whereas for the sclera, r^2^ were greater than 0.97. The permeation parameters were calculated, including permeation profile, flux (Jss, µg/h), permeability coefficient (Kp, cm/h), the cumulative permeated amount at 24 h (Cum abricitinib 6 h, µg), and theoretical plasma concentration in humans at the steady-state (Css, ng/mL), of baricitinib. Figure 7 shows the behavior of both liposomes on the sclera and Figure 8 shows the behavior of both liposomes on the cornea.

**Figure 9:**
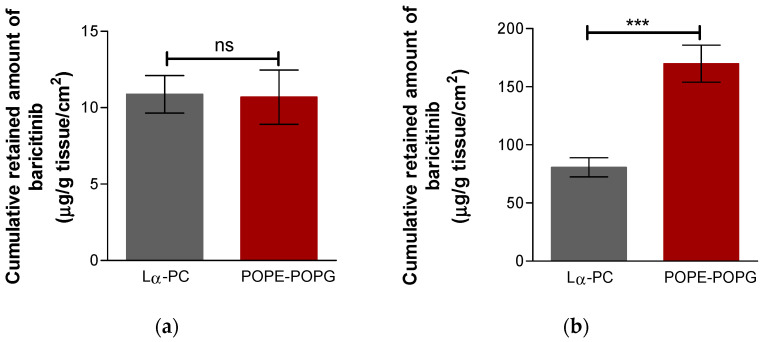
Baricitinib amount retained in the tissues: (**a**) cornea (**b**) sclera. Results are expressed as mean ± SD (n = 5). Lα-PC = L-α-phosphatidylcholine; POPE:POPG = 1-palmitoyl-2-oleoyl-phosphatidylethanolamine: 1-Palmitoyl-2-oleoyl-sn-glycerol-3-phosphoglycerol (3:1, mol/mol). *t*-Test analysis with a statistically significant difference: *** = *p* < 0.0001; ns = non-significant.

The baricitinib retained in the sclera and cornea is shown in Figure 9, both liposomes have significant differences in the sclera, but no statistical differences were found in the amount of baricitinib retained in the cornea. POPE:POPG retained baricitinib in the scleral tissue two-fold compared to Lα-PC, whereas baricitinib was retained equally by both liposomes in corneal tissue.

### 3.5. In Vitro Tolerance Study

#### 3.5.1. HET-CAM

**Figure 10:**
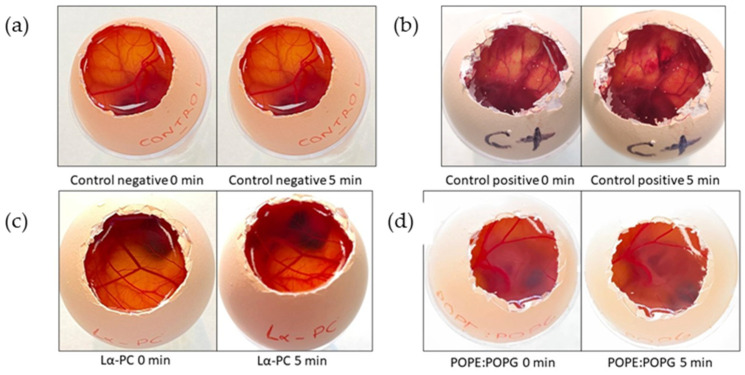
Evaluation of the irritant effect of the formulations by HET-CAM. (**a**) negative control (saline solution), (**b**) positive control (sodium hydroxide solution 0.1 N, (**c**) Lα-PC liposome (L-α-phosphatidylcholine), and (**d**) POPE:POPG liposome (1-palmitoyl-2-oleoyl-phosphatidylethanolamine: 1-Palmitoyl-2-oleoyl-sn-glycerol-3-phosphoglycerol (3:1, mol/mol).

The potential irritant effect of the formulations on the eyes was evaluated by the HET-CAM method, which consisted of applying the liposomes on the chorioallantoic membrane of 10-day embryonated eggs. No hemorrhaging, coagulation, or vessel lysis were observed 5 min after the application of the formulations (Figure 10). In contrast, hemorrhaging was observed in the positive control from the very beginning of applying the control, and after 5 min both hemorrhaging and coagulation were observed.

The values of the IS obtained after the HET-CAM test are reported in Table 7. IS values obtained for both liposomes were below 0.9, indicating that the liposomes did not show any irritating potential.

#### 3.5.2. Cornea Transparency

**Figure 11:**
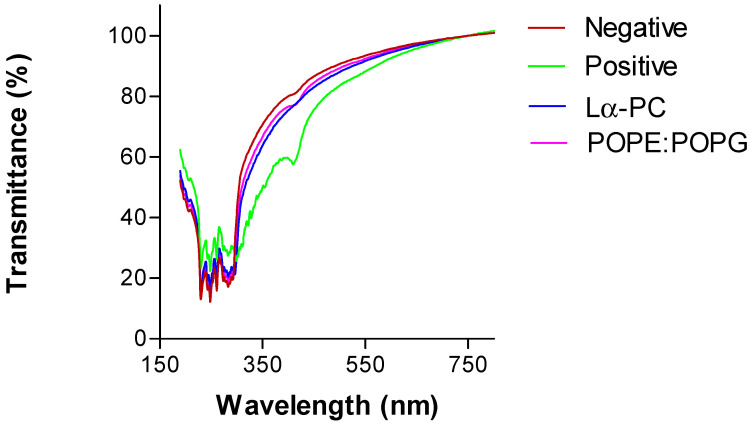
Transmittance from 190 to 850 nm wavelength of the corneas treated with PBS (negative control), ethanol (positive control), Lα-PC (L-α-phosphatidylcholine), and POPE:POPG liposomes (Lα-PC = L-α-phosphatidylcholine; POPE:POPG = 1-palmitoyl-2-oleoyl-phosphatidylethanolamine: 1-Palmitoyl-2-oleoyl-sn-glycerol-3-phosphoglycerol (3:1, mol/mol) after 10 min of incubation.

The transparency of the cornea was observed to assess any potential irritant effect of the liposomes on the cornea. Corneas treated with ethanol (positive control) show a decrease in the transmittance of up to 20% within the wavelengths 300–650 nm compared with the negative control, indicating a reduction of the transparency of the cornea. In contrast, the transmittance profile of both liposomes overlaps the negative control indicating that they do not affect the cornea transparency (Figure 11).

### 3.6. Corneal and Scleral Histological Study

**Figure 12:**
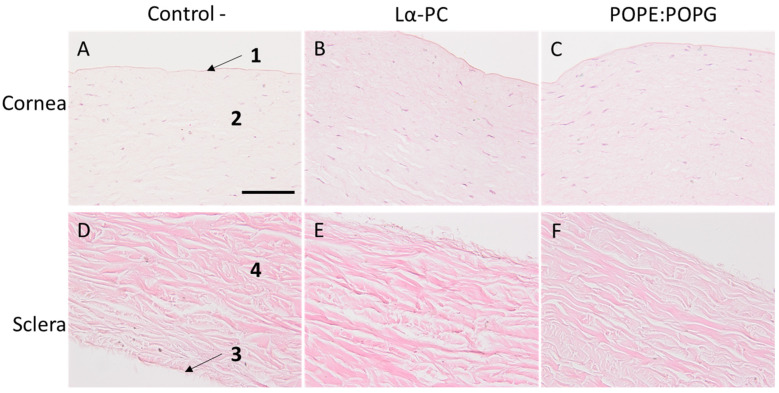
Sclera and cornea sections stained with hematoxylin and eosin. The upper row shows the cornea (**A**–**C**) and the sclera is shown below (**D**–**F**) in different conditions: control conditions (**A**,**D**); Lα-PC (**B**,**E**) and POPE:POPG (**C**,**F**); (1) corneal epithelium (non-keratinized stratified squamous epithelium); (2) substantia propria; (3) episclera; and (4) stroma. Magnification = 200×, scale bar = 100 µm.

Sclera and cornea did not show architectural alterations in the hematoxylin and eosin staining histological analysis in the control condition (Figure 12). The treatment with the two diluted liposomes did not alter either the cornea or the sclera.

## 4. Discussion

Two liposomes loading baricitinib were developed using two different lipids (Lα-P and POPE:POPG) with the aim of combining the effect of an immunomodulator and the effect of the lipids to supplement the tear film lipid layer. It is known that alterations in lipid composition, besides down-regulation in specific proteins or changes in the rheological behavior of the tears, are common in dry eye disease. Lipid-based formulations aim to mimic the tear film lipid layer by combining both components, the aqueous one and the lipid one [36]. As Hofauer and co-workers in a previous work had applied liposomal agents on buccal, nasal, and ocular mucosa in patients with Sjögrens’ syndrome to alleviate symptoms such as xerostomia, keratoconjunctivis sicca, and rhinitis sicca [21], and they had achieved positive results, it could be fruitful to load up liposomes with a specific immunomodulator, as it may result in synergistic effects, achieving an advance in the treatment.

Baricitinib has been used orally and no literature is available reporting nanostructured systems loading baricitinib for the topical route. It has been tested (only) once on the skin and there is very little information on formulating baricitinib in nano-systems. Bhaskarmurthy et al. [37] investigated the potential of baricitinib in reducing the inflammation in ear oedema TPA-induced inflammation in mice; we can underscore that they used a solution of baricitinib in acetone:DMSO, and they were testing on the skin, not on the mucosa or eye. Our study, therefore, encourages further research, building on the potential of baricitinib and its use in ocular mucosa. Both liposomes exhibit suitable characteristics for the ophthalmic application: their smallness in size will not damage the cornea upon application, and their pH is close to the tears’ value (7.4–7.5), so no irritation is expected; additionally, the osmolality was also within the criterium. The liposomes should resist aggregation due to Waals attraction forces because the zeta potential value is highly negative [38,39]. The surface electrical study focuses on two aspects. First, the dependence of the lipid composition, and secondly, the influence of the pH and ionic strength on ZP.

One of the major constraints of liposomal systems is the vesicle aggregation, with the concomitant destabilization of the system [40]. Moreover, the surface electrical charge of liposomes does play a fundamental role in the affinity on the corneal surface [41]. Along the same lines, the study of the surface electrical properties provides the expected relevant information; the lipid composition of liposomes has a direct influence on ZP, due to the different net charges of the lipids used: POPE:POPG liposomes showed much more negativity than Lα-PC liposomes. Another question is that the surface charge might also determine the interaction of liposomes with the ocular membrane due to the high negative charge of its mucins.

Absolute values of ZP showed a marked increase when the pH was increased (Figure 3). Changes in pH can affect the degree of lipid ionization. Specifically, the phosphate groups of the polar head of PC and PG are neutralized at acidic pH by the hydrogen ions of the dispersion medium. In contrast, as the pH value increased, that is, as the concentration of hydroxyl groups in the medium increased, a greater number of surface phosphate groups were dissociated. This determined the increase in the surface electric charge of these particles [42]. Nanosystems with ZP between +30 mV and −30 mV are considered to possess low stability [43]. The pH of tears is approximately 7.45 and ranges from 7.14 to 7.82, depending on diurnal and seasonal influences, or even 7.89 in dry eye patients with Sjögren’s disease [44]. Under these conditions, our liposomal formulations would have values outside the range +30 mV/−30 mV, which, predictably, will provide suitable stability [45].

The most frequently used aqueous vehicles in the preparation of eye drops are water for injection, isotonic sterile saline (SF), balanced salt solution (BSS^®^), and balanced salt solutions whose composition is similar to that of the internal ocular medium. Both the composition of these vehicles and physiologically buffer conditions mean that there are electrolytes of different valences that could influence the surface electrical characteristics of the liposomes. The electrolytes are mainly Na^+^, K^+^, Cl^−^, and HCO^−^, with lower levels of Mg^2+^ and Ca^2+^. In this way, the results of the electrokinetic analysis of the liposomes as a function of the ionic strength of the medium could be used to predict their stability in dispersion (aggregation tendency) and their mucoadhesive capacity.

The eye can tolerate tonicities within the equivalent range of 0.6–2% NaCl. However, to achieve isotonic solutions with tears, and to ensure that they are comfortable for the eye, an amount equivalent to 0.9% NaCl 0.15 M is generally used.

According to our results, shown in Figure 4, as the concentration of the salts increases the counterions accumulate closer to the particle surface, which compresses the double layer and weakens repulsive forces by reducing ZP [46].

Regarding the effect of AlCl_3_, ZP values were positive across the whole range, probably due to the adsorption of this ion into the surface of the liposomes. Finally, a high concentration of electrolytes equivalent to an isotonic solution might produce charge reversal or a large drop, showing slightly positive values. This fact will cause large size aggregates and rapid settling, which could give rise to a flocculated system that is easy to redisperse [47]. Additionally, thanks to their positive charge, they could interact better with the ocular film and prolong the residence time of the drug in the cornea, and they have increased the therapeutic interval [41,48].

A rapid release of baricitinib is observed within the first 4–6 h and it is followed by a slower drug release for both liposomes after these first six hours. Higher released amounts of baricitinib were obtained from the liposomes Lα-PC, about three-fold higher than the POPE:POPG liposomes, and this suggests that the lipid used in preparing the liposomes has a great impact on the extent of drug released. The release profile of both liposomes fitted to a one-site binding model corresponding to a hyperbola curve. The parameters of this model are Bmax, which corresponds to the maximum amount that can be released, and KD, which is the time needed to reach 50% of the drug released. Statistically significant differences were observed for Bmax. However, no statistical differences were found for KD, meaning that both liposomes release 50% of the drug within a similar time period (more or less in the first two and a half hours). Ansari et al. [49] developed polymeric nanoparticles loading baricitinib with the aim of improving baricitinib’s bioavailability in order to reduce the dose, and in turn, reducing the side effects. The authors prepared nanoparticles with poly-lactic-co-glycolic acid, and they conducted a deep characterization, obtaining an optimized formulation that exhibited sustained release over 24 h, which fitted the Higuchi model.

Despite the fact that liposome Lα- PC showed a higher in vitro drug release, this was not limiting for POPE:POPG in the penetration and permeation through the cornea, where the amount retained in the tissue was similar for both liposomes. It was POPE:POPG that showed higher permeation. In contrast, Lα- PC was superior to POPE:POPG in the permeation and penetration of baricitinib into and through the sclera. This is probably due to the difference in the composition of sclera and cornea, whereas the latter is mainly composed of type I collagen and proteoglycans, the sclera is primarily composed of connective tissue [50]. The amount of baricitinib retained in the corneal and scleral tissue would act as a reservoir and might enable a local anti-inflammatory and immunomodulatory effect without systemic side effects since the predicted plasma concentration at the steady-state (Css) is far below the concentration achieved in an oral administration [51].

## 5. Conclusions

The HET-CAM technique is an ideal model for testing ocular irritation since the chorioallantoic membrane is a highly vascularized structure and it is sensitive to chemicals such as the conjunctiva [52]. Both liposomes are supposed to be well-tolerated since no irritant potential was detected by the HET-CAM technique nor was any change observed in the histological analysis after the application of the liposomes on the corneal and scleral tissues. Additionally, no changes in the transparency of the cornea were observed either, meaning that the liposomes do not cause damage to the tissue exposed to them [34]. In light of these promising results, further studies should be carried out and looked at so as to better assess the efficacy of the liposomes in vivo. For instance, a dry eye in mice models would allow for the evaluation of the efficacy of the liposomes similarly to the work of Stevenson et al. [19,20], in which the researchers tested tofacitinib, a JAK inhibitor, applied topically in dry eye-induced mice, and they monitored cytokines expression obtaining excellent outcomes in reducing the ocular inflammation. Since the products intended for ophthalmic use should be sterilized, in this sense, future studies should also consider extruding the liposomes through a 0.22 µm pore size, as well as, investigating the effect of the number of lipid layers composing the liposome on the drug release. Another important point for future studies is the shelf-life of the formulations, and stability studies should also be performed.

Two liposomal formulations have been developed for ocular delivery intended for alleviating dry eyes related to Sjögren’s syndrome. The liposomes were prepared using two different lipids, Lα-PC and POPE:POPG, both loading baricitinib, a Janus kinase inhibitor. Lα-PC led to higher flux, permeation, and retention in the sclera, whereas POPE:POPG led to higher flux and permeation in the cornea. The formulations showed no irritant effects on the chorioallantoic membrane. Additionally, the liposomes did not affect the cornea transparency when applied and the histological analysis did not reveal any structural alteration. The two liposomes have shown promising results for ocular application, and further studies, such as in vivo tests, should be conducted to evaluate their efficacy, and hence, confirm their suitability in the management of dry eyes in Sjögren’s syndrome.

## Figures and Tables

**Table 1:** Experimental conditions for the in vitro release test.

Parameters	Conditions
Receptor fluid	Transcutol^®^ P
Cell volume	4.9 mL
Diffusion area	0.64 cm^2^
Membrane	Dialysis membrane
Replicates	5 replicates
Temperature	32 ± 0.5 °C
Stirring	500 r.p.m.
Dose	500 µL of liposomes (Lα-PC 13.99 μg/mL and POPE:POPG 9.83 μg/mL)
Sample volume	200 µL
Sampling times	0 (pre-sample time point), 3.0 h, 7.0 h, 20.3 h, 24.5 h, 28.3 h, and 31.0 h

Lα-PC = L-α-phosphatidylcholine; POPE:POPG = 1-palmitoyl-2-oleoyl-phosphatidylethanolamine: 1-Palmitoyl-2-oleoyl-sn-glycerol-3-phosphoglycerol (3:1, mol/mol).

**Table 2:** Experimental conditions for the ex vivo permeation test.

Parameter	Conditions
Receptor fluid	Transcutol^®^ P
Cell volume	4.9 mL
Diffusion area	0.64 cm^2^
Membrane	Cornea and Sclera
Replicates	5 replicates
Temperature	37 ± 0.5 °C or 32 ± 0.5 °C
Stirring	500 r.p.m.
Dose	500 µL of liposomes (Lα-PC or POPE:POPG)
Sample volume	200 µL
Sampling times	0 (pre-sample time point), 2.1 h, 4.2 h, and 6.0 h

Lα-PC = L-α-phosphatidylcholine; POPE:POPG = 1-palmitoyl-2-oleoyl-phosphatidylethanolamine: 1-Palmitoyl-2-oleoyl-sn-glycerol-3-phosphoglycerol (3:1, mol/mol).

**Table 3:** Chromatographic conditions for the determination of baricitinib.

Parameters	Conditions
Chromatographic column	Symmetry C18 (4.6 × 75 mm, 3.5 µm)
Mobile phase	Ammonium Formate 10 mM pH 7:I (75:25 *v*/*v*)
Flux	1 mL/min
Injection volume	10 μL
Wavelength	Ex 310 nm and Em 390 nm
Standard concentrations range	0.031 to 1 μg/mL

**Table 4:** pH, osmolality, and encapsulation efficiency (EE) of the liposomes. Results are expressed by mean ± SD (n = 3).

Liposome	pH	Osmolality (mOsm/Kg)	EE (%)
Lα-PC	7.4 ± 0.1	306 ± 2	12 ± 0.9
POPE:POPG	7.4 ± 0.1	305 ± 5	11 ± 1.1

Lα-PC = L-α-phosphatidylcholine; POPE:POPG = 1-palmitoyl-2-oleoyl-phosphatidylethanolamine: 1-Palmitoyl-2-oleoyl-sn-glycerol-3-phosphoglycerol (3:1, mol/mol).

**Table 5:** Liposome composition with their respective hydrodynamic diameter, polydispersity index (PDI), and zeta potential (ZP). Results are expressed by mean ± SD (n = 3).

Composition	Formulation	Hydrodynamic Diameter (nm)	PDI	Zeta Potential (mV)
Lα-PC	blank	73.0 ± 3.0	0.040	−22.5 ± 1.1
	+baricitinib	61.7 ± 0.5	0.081	−20.7 ± 0.5
POPE:POPG	blank	60.5 ± 0.6	0.124	−32.0 ± 3.0
	+baricitinib	51.7 ± 0.8	0.202	−37.0 ± 5.0

Lα-PC = L-α-phosphatidylcholine; POPE:POPG = 1-palmitoyl-2-oleoyl-phosphatidylethanolamine: 1-Palmitoyl-2-oleoyl-sn-glycerol-3-phosphoglycerol (3:1, mol/mol).

**Table 6:** Best fit values in the kinetic modeling for the liposomal formulations and the statistical analysis by a *t*-Test between Lα-PC and POPE:POPG. Significance level set at *p* < 0.05.

Parameters	Lα-PC	POPE:POPG
Bmax (µg)	21.42	6.836
KD (h)	2.735	2.398
SE Bmax	0.424	0.052
SE KD	0.290	0.105
95% CI Bmax	20.33 to 22.51	6.701 to 6.971
95% CI KD	1.989 to 3.480	2.128 to 2.668
R^2^	0.9992	0.9999
*p*-value Bmax	<0.0001	
*p*-value KD	0.3047	

Lα-PC = L-α-phosphatidylcholine; POPE:POPG = 1-palmitoyl-2-oleoyl-phosphatidylethanolamine: 1-Palmitoyl-2-oleoyl-sn-glycerol-3-phosphoglycerol (3:1, mol/mol). Bmax = maximum amount released (µg); KD = time required to reach 50% of the drug release (h); SE = standard error; CI = confidence interval.

**Table 7:** Irritation score (IS) of the liposomes tested by the HET-CAM technique.

Formulation	Irritation Score (IS)	Classification
Lα-PC	0.03	Non-irritating
POPE:POPC	0.02	Non-irritating

IS ≤ 0.9, non-irritating/slightly irritating; 0.9 < IS ≤ 4.9, moderately irritating; 4.9 < IS ≤ 8.9, irritating; and 8.9 < IS ≤ 21, severely irritating [26]. Lα-PC = L-α-phosphatidylcholine; POPE:POPG = 1-palmitoyl-2-oleoyl-phosphatidylethanolamine: 1-Palmitoyl-2-oleoyl-sn-glycerol-3-phosphoglycerol (3:1, mol/mol).

## Data Availability

The data presented in this study are available on request from the corresponding author.
